# Adieu

**Published:** 1881-09

**Authors:** B. M. Wilkerson


ADIEU.—Wishing our senior editor abundant success with his "other duties, present and prospective," we reluctantly say to him, *vive*, *vale*, and ask his friends to be our friends, with the assurance that we shall continue to "press onward and upward."

B. M. WILKERSON.

